# The Prognosis of Blunt Bowel and Mesenteric Injury—The Pitfall in the Contemporary Image Survey

**DOI:** 10.3390/jcm8091300

**Published:** 2019-08-24

**Authors:** Chien-Hung Liao, Feng-Jen Hsieh, Chih-Chi Chen, Chi-Tung Cheng, Chun-Hsiang Ooyang, Chi-Hsun Hsieh, Shang-Ju Yang, Chih-Yuan Fu

**Affiliations:** 1 Department of Trauma and emergency surgery, Chang Gung Memorial Hospital, Chang Gung University, 5 Fuhsing St., Taoyuan 333, Taiwan; 2 Department of Physical Medicine and Rehabilitation, Chang Gung Memorial Hospital, School of Medicine, Chang Gung University, 5 Fuhsing St., Taoyuan 333, Taiwan

**Keywords:** blunt bowel injury, blunt mesenteric injury, computed tomography, prognosis

## Abstract

Delayed diagnosis and intervention of blunt bowel and mesenteric injury (BBMI) is a hazard because of poor prognosis. Computed tomography (CT) is the standard imaging tool to evaluate blunt abdominal trauma (BAT). However, a high missed diagnosis rate for BMMI was reported. In this study, we would like to evaluate the presentation of CT in BBMI. Moreover, we want to evaluate the impact of deferred surgical intervention of BBMI on final prognosis. We performed a retrospective study from 2013–2017, including patients with BAT and BBMI who underwent surgical intervention. We evaluated clinical characteristics, CT images, and surgical timing, as well as analyzed the prognosis of BBMI. There were 6164 BAT patients and 188 BMI patients included. The most common characteristics of CT were free fluid (71.3%), free air (43.6%), and mesenteric infiltration (23.4%). There were no single characteristics of a CT image that can predict BBMI significantly. However, under close monitoring, we find that deferred intervention did not prolong the hospital and intensive care unit stays and did not worsen the prognosis and mortality.

## 1. Introduction

Blunt bowel and mesenteric injury (BBMI) is one of the most difficult challenges in the trauma setting [1,2,3]. Acute hemorrhage from BBMI will lead lethal loss of blood volume, and the breakdown of the integrity of the GI tract will induce splint of bowel content, progression of bacterial contamination and predisposing sepsis occurrence [4]. The reported incidence of BBMIs among abdominal trauma patients is 1% to 3% and resulted in morbidity and mortality among trauma patients [5,6,7].

Currently, computed tomography (CT) scans are commonly performed for hemodynamically stable abdominal trauma patients during evaluation [8,9,10]. Because of the improvement of the diagnostic modality and care quality, the treatment algorithms of blunt truncal trauma weighted toward conservative management amplify the need to identify bowel and mesenteric trauma early in the treatment process. Most injuries are readily detected, but others are more subtle and require careful evaluation and interpretation. However, an exploratory laparotomy for all the patients suspected of having BBMI leads to a high negative rate, and as a consequence waste medical cost and hospitalization [1,11]. Several authors advised on the indication and condition that non-operative management (NOM) can be applied to patients with BBMI. However, the prognosis and cost of deferred management of BBMI are still controversial [12,13,14,15]. This dilemma in diagnosis makes the diagnostic rate of BBMI more challenging than before.

In this study, we would like to evaluate the characteristics of the CT image to detect the BBMI. Moreover, we want to evaluate if the risk of deferred surgical intervention of BBMI will worsen the prognosis or not.

## 2. Experimental Section

### 2.1. Materials and Methods

We conducted a prospective data collection trauma registry in Chang Gung Memorial Hospital (CGMH), Linkou, Taiwan. Demographic data, medical, perioperative and hospital course, follow-up, and information regarding complications were recorded into a computerized database prospectively. We performed a retrospective review of all patients suffered from blunt bowel and mesenteric injury (BBMI) from January 2010 to December 2017 in Chang Gung Memorial Hospital, Linkou, which is a Level I trauma center in Taiwan. The study was approved by the Internal Review Board of CGMH, IRB No.: 201900304B0.

### 2.2. Study Population

**Figure 1:**
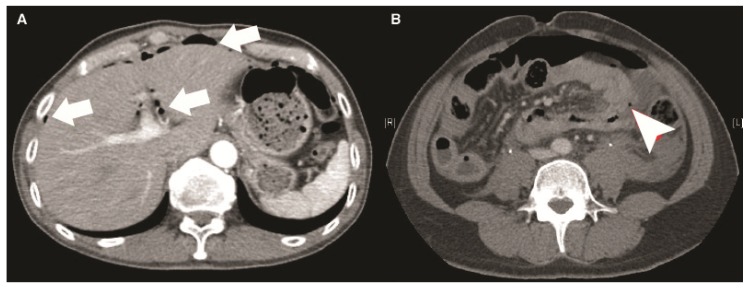
(**A**) The axial computed tomography presented multiple extraluminal air (arrows). (**B**) The abdominal computed tomography revealed a small extraluminal air bubble with focal fluid (arrowhead).

**Figure 2:**
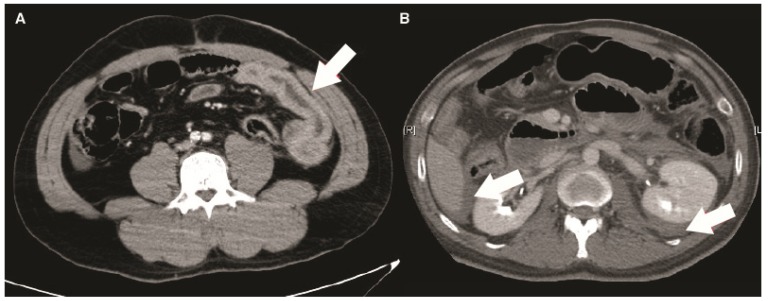
(**A**) The axial computed tomography revealed an abdominal bowel wall with thickened, hyperemic and fluid-filled bowel loops (arrow). (**B**) The image showed multiple intraperitoneal and retroperitoneal free fluid (arrows) without solid organ injury, which imply the possibility of blunt bowel and mesenteric injury.

**Figure 3:**
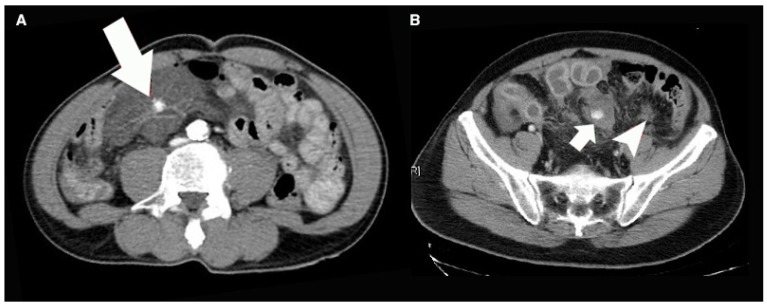
(**A**) The axial contrast-enhanced abdominal computed tomography showed the mesenteric injury with focal hematoma and mesenteric pseudoaneurysm (arrow). (**B**) The axial contrast-enhanced computed tomography revealed multiple mesenteric lacerations present as mesenteric extravasation (arrow) and mesenteric infiltration (arrowhead).

All patients were managed by the trauma team from emergency bay arrival to discharge. All the management were under the same clinical protocol. Demographic information was collected with the results of physiological and biochemical data and image finding. Besides, the injury severity score (ISS), anatomic injury scale of abdomen (AIS abdomen), reverse trauma score (RTS), trauma score—injury severity score (TRISS), surgical finding, and associated injury were recorded. For patient with overt abdominal evisceration, hemodynamic unstable or severe peritoneal signs, we arranged surgical intervention immediately. For patients with stable hemodynamics and without peritoneal signs, we will arrange contrast-enhanced computed tomography (CT) for further bowel and mesenteric evaluation if BBMI was suspected. All the CT images were reviewed by experienced trauma surgeons and radiologists to collect the presence of characters of BBMI pattern in CT, including bowel wall discontinuity, extraluminal air (Figure 1), bowel wall thickening (Figure 2), abnormal bowel wall enhancement (Figure 2), intraperitoneal free fluid (Figure 2), mesenteric extravasation (Figure 3), mesenteric vascular bleeding (Figure 3), mesenteric pseudoaneurysm (Figure 3), mesenteric infiltration (Figure 3), and abdominal wall injury were recorded [16,17].

For the operative timing, we divided our patients into two groups. Patients who had an operation within 24 h since the emergency department (ED) arrival were included in the early group and patients who had an operation after more than 24 h were included in the deferred group. The postoperative recovery, complication, the length of intensive care unit stays (ICU LOS), the length of hospital stays (HLOS), and presence of mortality were collected.

### 2.3. Statistical Analysis

Pearson χ^2^ test and Fisher’s exact test were used as appropriate to compare categorical variables. Quantitative variables were compared by Student’s t test. The odds ratio and 95% confidence interval (CI) were calculated by logistic regression in the case of qualitative variables and linear regression for quantitative ones. Statistical analysis was done with SPSS v 20.0 for Macintosh (SPSS Inc, Chicago, IL, USA). A value of *p* < 0.05 was considered statistically significant.

## 3. Results

**Figure 4:**
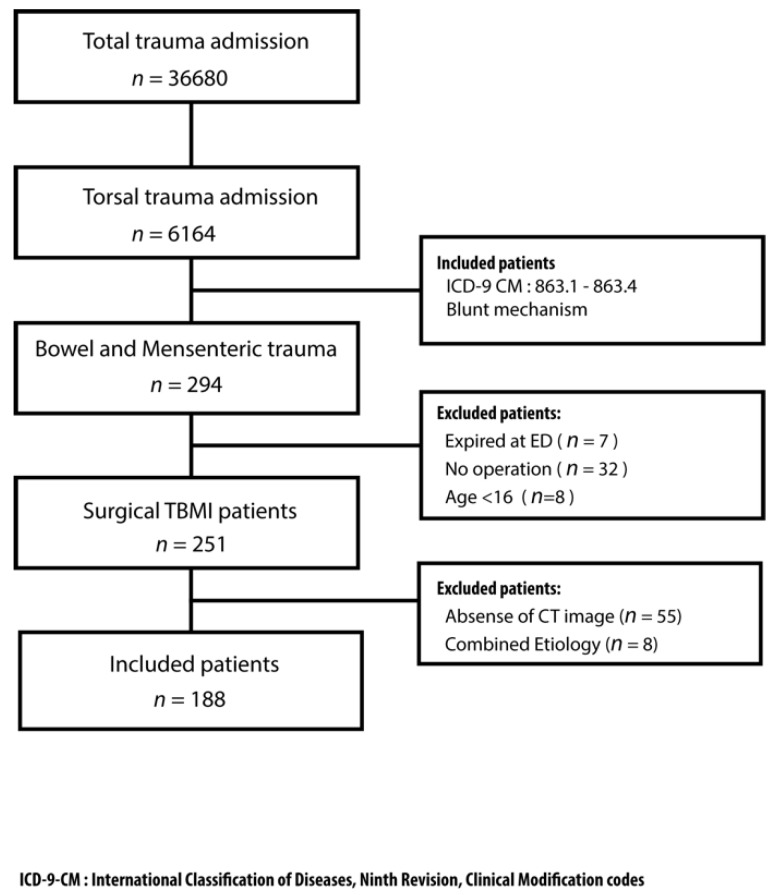
The flow chart of this study.

In total, 204,441 patients visited the trauma bay, and 36,680 patients required further admission. There were 6164 patients with blunt thoracoabdominal trauma who required admission to the trauma ward during the study period. Figure 4 presents the flow of this study.

The cohort consisted of 188 patients, with 146 males (77.7%) and 42 females (22.3%) as Table 1 showed. The incidence of BBMI was 3.05% of blunt torsal trauma admissions. The median age was 45 years, with an interquartile range from 29 to 59 years. The most common mechanism was motor vehicle collision, followed by falling. The mean ISS was 17.2 ± 11.2. The ICU LOS was 6.5 ± 8.5 days, and the mean HLOS was 17.3 ± 15.6 days. The demographic data for the patients with BBMI is summarized in Table 1. For the Surgical BBMI, five patients (2.7%) had bowel wall discontinuity, eighty-two patients had extraluminal air (43.6%), 43 patients had bowel wall thickening (22.9%), and 23 patients had abnormal bowel wall enhancement (12.2%). Furthermore, 27 patients had mesenteric extravasation (14.4%), 4 had mesenteric vascular bleeding (2.1%), 44 patients had mesenteric infiltration (23.4%), and 23 patients had mesenteric pseudoaneurysm (12.2%). One hundred and thirty-four patients had intraperitoneal and retroperitoneal fluid collection (71.3%) and 14 patients had abdominal wall injury (7.4%) by contrast extravasation during CT examination. There were still 12 patients (6.6%) had no specific finding in abdominal CT. Nine of the patients were in the early group. The nine patients that presented the incidental finding underwent a laparotomy for associated solid organ injuries. Three patients of the delayed group had repeat CT scans (48 h, 56 h, and 144 h), and after finding the abnormal presentation we did the operation. Thirty-nine patients had contemporary solid organ injury during surgical exploration. Nine patients underwent transarterial embolization (TAE) (4.8%). Five patients underwent TAE because of solid organ injury, including one suspected mesenteric injury and four patients who underwent TAE because of a pelvic fracture. As Table 1 presents, there were 58 mesenteric injury, 125 bowel injury, and 5 combined mesenteric and bowel injuries. One hundred and three patients needed a second operation as the definite operation after a primary damage control laparotomy. Fifty patients had a bowel resection and only 6 patients needed stoma creation. In this series, 161 patients had early diagnosis and surgical intervention within 24 h (85.6%), and there were 27 patients with deferred diagnosis (14.4%).

By dividing the operative timing as in Table 2, we found mesenteric extravasation and mesenteric hematoma were predominant in the early surgical group compared with the deferred group. The surgeons might be alerted by the sign of extravasations of the mesentery to intervention. However, the presence of TAE was higher in the deferred group (14.8%) than the early group (3.1%). The ISS, AIS abdomen, RTS, TRISS, ICULOS, and HLOS were comparable between both groups. The necessity of a 2nd operation, bowel resection, and stoma creation were similar in both groups. Although the intraabdominal infection rate was higher in the deferred group (25.9%) than early group (10.6%), there was no statistical significance (*p* = 0.054). The mortality rate was similar between both groups, too (early group 10.5% vs. deferred group 11.1%, *p* = 1.000).

Twenty-one patients died with their BBMI (11.2% mortality). There were 11 patients who suffered from hemodynamic instability and an RTS below six. Another six patients suffered from severe associated cerebral injury and there were three patients that were of old age (>80 years) and had decompensated cardiopulmonary functioning. One patient suffered from severe nosocomial pneumonia and intrabdominal infection. Four of the deceased patients had intraabdominal infection (IAI): two patients showed apparent bilious content from drainage but died in days due to severe trauma; one had a stoma creation but still suffered from partial leakage from the peristomal area, whose IAI recovered properly after conservative management, however cerebral failure lead mortality. Another one developed severe sepsis related to an anastomosis leak, IAI, and nosocomial pneumonia. Compared with the survival cases, the occurrence of typical CT image characters of BBMI were similar. ISS was higher in the deceased group (29.33 ± 10.07) than survival group (15.65 ± 10.43) with statistical significance (*p* < 0.001) as Table 3 presents.

## 4. Discussion

In this study, we evaluated the typical characteristics in CT initially of the patient who underwent a surgical approach for BBMI. We found that single image characteristics did not show a good predictive value in detecting BBMI. Meanwhile, although there is a high delayed diagnostic rate of BBMI, deferred operation (>24 h) from ED arrival seems not worsen the prognosis of this trauma challenge. A CT scan of the abdomen has become the gold standard diagnostic modality for the hemodynamically stable blunt trauma patient for parenchymal organs, however the performance for BBMI were still overlooked. In the previous literature, there were ten common characteristics advised while evaluating the CT, where the most important one is to select out the surgical BBMI [16,18]. The typical image patterns of BBMI cannot show both good sensitivity and specificity properly. However, these methods of diagnosing BBMI are suboptimal with a high false negative rate of 12–13% [1,19]. While operating on every abnormal CT scan result in blunt trauma patients would ensure no missed injuries, this is not a practical option because it would increase morbidity, cost, and length of stay (LOS). The surgeon must weigh the risks of the negative laparotomy versus the risk of missed injury in formulating a treatment plan for BBMI [20].

Because of the increase in knowledge about this injury, we applied NOM with close monitoring in patients with characteristics of BBMI from CT, and we took action once the situation became disoriented. Despite there being the obvious presentation of BBMI, NOM with the final observation might prevent the negative laparotomy rate and preserve the life as far as possible.

Delayed diagnosis and surgical intervention of BBMI had been thought to worsen the prognosis and increase the mortality rate [4,5,6,14,15]. In the age of non-operative management predominant in trunk trauma, this thought had been challenged. In this study, we figured out the deferred group for BBMI patients, whether intentional or not, and the prognosis was similar to the early group. In this series, the delay between ED to treatment in the delayed group is from 24 to 144 h with a median 27 h. The post-operative infection and intraabdominal infection rate were similar than the early group. The HLOS and ICU LOS were also not being prolonged in the deferred group. The mortality of BBMI was still related to the ISS score [21]. To handle other associated lethal problems first, if there was no active hemorrhage injury in BBMI, can stabilize the patient’s hemodynamics, after which we can then perform the surgical intervention to BBMI for bowel repair or section, which might prevent the patients under operation being in the lethal triad. In our deferred group, we also found a high possibility of TAE for associated injuries. BBMI is frequently found in patients who undergo laparotomy for hemostasis of parenchyma organ injury, but chances to directly diagnose BBMI via laparotomy are decreasing as TAE is becoming more popular. That might explain why we might defer the surgical intervention to BBMI. Several literatures support that diagnostic delays might not worsen the prognosis of BBMI, which might support our finding differently [15,20,22,23,24,25]. We agree that diagnostic delays should be avoided to minimize morbidity and mortality of BBMI. However, under close monitoring, we can prevent unnecessary laparotomy and minimize the morbidity and mortality of BBMI too. In our institute, we handle all the trauma patients from ED arrival. For the patients with a suspicion of BBMI, we will keep observation for 24 h. In the case of blunt chest trauma patients with pneumoperitoneum, to diagnose the BBMI becomes more complicated. In this situation, we did observations if there were any toxic pattern of BBMI presented. Once the patients present peritoneal signs or experience clinical disorientation, we did surgical intervention, whether laparotomy or laparoscopy. For the unconscious patients, we apply the same policy too. In this study, we performed 15 negative laparotomies because we initially intended to diagnose BBMI. Changes in clinical and laboratory findings (i.e., unexplained tachycardia, leukocytosis) may be used in the decision-making process. It may also be reasonable to recommend routine repeated CT scans at 12 h or 24 h for these patients [22], although the effectiveness of such a strategy has yet to be demonstrated [7].

In case of an undetermined BBMI, the surgeon-based evaluation should be considered. Such cases warrant surgical evaluation for possible admission and serial examination by the surgical team [7,23]. They concluded that this is particularly important for non-surgeons initially involved in the care of trauma patients. The value of having the trauma surgeon review the CT images and the patient’s condition is another consideration to improve the diagnosis of BBMI [7,16].

A predictive score for BBMI was developed, which is the easier index to predict the presence of BBMI in the blunt trauma patient [1], but the results seem still not confirmed properly [3,24]. However, the individual components of the index are not reliable as well. Because abdominal pain is a subjective factor that might shift in different patients who have different perspectives. Some studies show that physical examination findings did not correlate with BBMI significantly [19] and cited a sensitivity and specificity of 53% and 69%, for the physical examination diagnosis of BBMI [25]. The elevated white blood cell count (WBC) is an indicator for BBMI also, however it is also an acute phase marker of inflammation which trauma can induce without other injury takes place. Therefore, it might not be specific to the BBMI. There were three components needed to be checked out: The presence of hemorrhage, the integrity of the hollow organ, and the integrity of blood supply from the mesenteric to the bowel.

There are several limitations to this study. First, this was a retrospective study, and the selection of the patients could not be randomized. Although all of the data were collected prospectively, and the characteristics of the patients were similar and homogeneous, selection bias could not be prevented entirely. Another obvious limitation of this study is that the sample size was a small and unsymmetrical distribution, which consequently limited the power to detect statistical significance. Third, there were still numerous patients who underwent surgical intervention for BBMI in this period because of clinical judgment that we did not have an image to review, which will result in another selective bias. Malhotra et al. [26] presented multiple findings in CT that show a high probability to detect BBMI. However, because we showed limited data about this aspect, we cannot perform further validation for this concept in the current study.

## 5. Conclusions

In this study, we figured out the poor predictive value of single characteristics of image patterns of abdominal CT to determinate BBMI. A total of 6.6% of patients without characteristics of CT might take place. However, the deferred operation of BBMI seems not to prolong the ICU LOS or HLOS, nor increase mortality.

## Figures and Tables

**Table 1:** The characteristics of patients with blunt bowel and mesenteric injury.

Characteristics	
Total Numbers	188
Age (Mean ± SD)	45.3 ± 18.3
Male Gender (*n*, %)	146 (77.7%)
ISS (Mean ± SD)	17.2 ± 11.2
RTS (Mean ± SD)	7.078 ± 1.394
TRISS (Mean ± SD)	0.871 ± 0.240
CT Characters	
Bowel Wall Discontinuity (*n*, %)	5 (2.7%)
Extraluminal Air (*n*, %)	82 (43.6%)
Bowel Wall Thickening (*n*, %)	43 (22.9%)
Abnormal Bowel Wall Enhancement (*n*, %)	23 (12.2 %)
Mesenteric Extravasation (*n*, %)	27 (14.4%)
Mesenteric Vascular Bleeding (*n*, %)	4 (2.1%)
Mesenteric Infiltration (*n*, %)	44 (23.4%)
Mesenteric Pseudoaneurysm (*n*, %)	23 (12.2%)
Intraperitoneal and Retroperitoneal Free Fluid (*n*, %)	134 (71.3%)
Abdominal Wall Injury (*n*, %)	14 (7.4%)
Surgical timing	
Early Operation, <24 h (*n*, %)	161 (85.6%)
Deferred Operation, >24 h (*n*, %)	27 (14.4%)
Surgical finding	
Mesenteric Injury	58 (30.9%)
Bowel Injury	125 (66.5%)
Combined Injury	5 (2.6%)
ICULOS (days, Mean ± SD)	6.5 ± 8.5
HLOS (days, Mean ± SD)	17.3 ± 15.6
Mortality (*n*, %)	21 (11.2%)

SD: Standard deviation; ISS: Injury severity score; RTS: Reverse trauma score; TRISS: Trauma Score—Injury Severity Score; CT: Computed tomography; ICU LOS: The length of intensive care unit stays; HLOS: The length of hospital stays.

**Table 2:** The comparison of early and deferred diagnosis groups.

Characteristics	Early Diagnosis *n* = 161	Deferred Diagnosis *n* = 27	*p* Value
Age	26.1 ± 17.4	40.4 ± 22.5	0.224
ISS	17.2 ± 11.2	17.6 ± 10.5	0.718
AIS abdomen	2.9 ± 0.7	3.0 ± 0.6	0.437
RTS	7.03 ± 1.46	7.35 ± 0.89	0.129
TRISS	0.86 ± 0.25	0.92 ± 0.17	0.289
Bowel Wall Discontinuity	4 (2.5%)	1 (3.7%)	0.569
Extraluminal Air	70 (43.5%)	12 (44.4%)	0.466
Bowel Wall Thickening	36 (22.4%)	7 (25.9%)	0.535
Abnormal Bowel Wall Enhancement	22 (13.7%)	1 (0.5%)	0.072
Mesenteric Extravasation	27 (16.8%)	0 (0%)	0.008 *
Mesenteric Vascular Bleeding	3 (1.9%)	1 (3.7%)	0.489
Mesenteric Infiltration	40 (24.8%)	4 (14.8%)	0.142
Mesenteric Pseudoaneurysm	23 (14.3%)	0 (0%)	0.017 *
Intraperitoneal and Retroperitoneal Fluid	116 (72.0%)	18 (66.7%)	0.453
Abdominal Wall Injury	11 (6.8%)	3 (11.1%)	0.366
TAE	5 (3.1%)	4 (14.8%)	0.026 *
Mesentery Injury	53 (32.9%)	5 (18.5%)	0.133
Bowel Injury	103 (64.0%)	22 (81.5%)	0.075
Combined Injury	5 (3.1%)	0 (0%)	
Necessity for 2nd Operation	88 (54.7%)	15 (55.6%)	1.000
Bowel Resection	42 (26.1%)	8 (29.6%)	0.814
Stoma Creation	4 (2.5%)	2 (7.4%)	0.207
Intrabdominal Infection	17 (10.6%)	7 (25.9%)	0.054
ICU LOS	6.37 ± 8.4	7.04 ± 8.8	0.718
HLOS	17.1 ± 15.4	18.3 ± 16.6	0.724
Mortality	18/161 (11.2%)	3/27 (11.1%)	1.000

ISS: Injury severity score; RTS: Reverse trauma score; TRISS: Trauma Score—Injury Severity Score; AIS abdomen: Anatomic injury scale of abdomen; ICU LOS: The length of intensive care unit stays; HLOS: The length of hospital stays. *: *p* < 0.05 with a statistical significance.

**Table 3:** The comparison between survival and deceased groups in blunt bowel and mesenteric injury.

Characteristics	Survival *n* = 167	Deceased *n* = 21	*p* Value
Age	44.38 ± 17.59	53.33 ± 21.13	0.060
ISS	15.65 ± 10.43	29.33 ± 10.07	<0.001 *
RTS	7.29 ± 1.10	5.43 ± 2.24	0.001 *
TRISS	0.91 ± 1.86	0.56 ± 0.37	<0.001 *
AIS abdomen	2.90 ± 0.65	3.29 ± 0.90	0.069
Bowel Wall Discontinuity	5 (3.0%)	0 (0%)	1.000
Extraluminal Air	75 (44.9%)	7 (33.3%)	0.358
Bowel Wall Thickening	38 (22.8%)	5 (23.8%)	1.000
Abnormal Bowel Wall Enhancement	1 (4.8%)	2 (13.2%)	0.479
Mesenteric Extravasation	23 (13.8%)	4 (19.0%)	0.512
Mesenteric Vascular Bleeding	3 (1.8%)	1 (4.8%)	0.380
Mesenteric Infiltration	37 (22.2%)	7 (33.3%)	0.277
Mesenteric Pseudoaneurysm	19 (11.4%)	4 (19.0%)	0.297
Intraperitoneal and Retroperitoneal Fluid	119 (71.3%)	15 (71.4%)	1.000
Abdominal Wall Injury	11 (6.6%)	3 (14.3%)	0.194
Transcatheter Arterial Embolization	6 (3.6%)	3 (14.3%)	0.065
Early Operation	143 (85.6%)	18 (85.7%)	1.000
Intraabdominal Infection	19 (11.4%)	5 (23.8%)	0.155

ISS: Injury severity score; RTS: Reverse trauma score; TRISS: Trauma Score—Injury Severity Score; AIS abdomen: Anatomic injury scale of abdomen. *: *p* < 0.05 with a statistical significance.
